# Knowing Your Accessory Foot Ossicles and Avoiding Misdiagnoses: A Case Report of Painful Os Vesalianum Pedis

**DOI:** 10.7759/cureus.27380

**Published:** 2022-07-27

**Authors:** Miguel De Castro Correia, Tiago Rodrigues Lopes

**Affiliations:** 1 Physical Medicine and Rehabilitation, North Rehabilitation Center, Vila Nova de Gaia, PRT

**Keywords:** lateral foot pain, acessory ossicles, pseudo-jones fracture, jones fracture, os vesalianum pedis

## Abstract

Os vesalianum pedisis located proximal to the base of the fifth metatarsal. Rarely, this accessory ossicle can be the source of lateral foot pain. There are very few cases of symptomatic os vesalianum pedisdescribed in the literature, and most of them were surgically managed. We report a painful case of os vesalianum pedis managed conservatively. A 25-year-old professional soccer player presented with lateral left midfoot pain. There was no known acute sprain or trauma, and no history of injuries in the left lower limb. The athlete reported both mechanical and inflammatory pain findings and tenderness on the palpation of the fifth metatarsal base. We conducted a radiographic study of the left foot and found an image compatible with os vesalianum pedis​​​​​​​. A right foot X-ray was also performed, and similar findings were reported, although the athlete had no pain. The athlete was treated conservatively, and the return-to-play was seven days.

Due to the unspecific symptoms of our athlete, many diagnoses could be considered such as peroneus brevistendinopathy, lateral plantar fasciitis, ligamentum plantare longumsprain. However, the X-ray findings led us to other possible pathologies, mainly affecting the bone. Integrating clinical and radiological findings is mandatory to achieve a proper diagnosis and avoid mistakenly diagnosing a fracture such as a Jones fracture or pseudo-Jones fracture. Even though os vesalianum pedis​​​​​​​ is usually asymptomatic, this condition can lead to chronic pain. Well-designed conservative management should always be pursued to treat this condition as it might prevent the need for surgery.

## Introduction

The foot and ankle are very prone to anatomic variations, either accessory ossicles, bipartitions, or coalitions [1]. According to a recent study, the prevalence of foot accessory ossicles can be as high as 26% [2]. Os vesalianum pedis was first illustrated in the 16th century [2] and named after its illustrator, Vesalianum, in the 20th century [3]. This accessory ossicle is located proximal to the base of the fifth metatarsal within the peroneus brevis tendon [1]. Its prevalence is usually between 0.1% and 0.5%, but some series report prevalence as high as 6% [4-6]. Even though some authors often describe it as bilateral [7], a radiographic study including more than 1500 radiographs, didn't find any patient with bilateral os vesalianum pedis [1]. Similar to other accessory ossicles, os vesalianum pedis is mainly asymptomatic and found incidentally on radiographs, usually after an acute injury [7]. However, in a few patients, this accessory ossicle can be the source of lateral foot pain [1]. The differential diagnosis of this condition is the Jones fracture (a fracture through the metaphyseal-diaphyseal junction of the fifth metatarsal); pseudo-Jones fracture (fifth metatarsal proximal tubercle avulsion); and in the pediatric population, Iselin’s disease (fifth metatarsal traction apophysitis) and the mere presence of the growth plate. There are very few cases of symptomatic os vesalianum pedis in the literature-the first one being described in 1957 [8]-and most of them were surgically managed due to the chronicity of symptoms (ossicle excision and peroneus brevis tenorrhaphy or reinsertion) [7-12]. We report the case of a painful os vesalianum pedis in a male professional soccer athlete with full symptom resolution after conservative management.

## Case presentation

A 25-year-old male professional soccer player presented with lateral left midfoot pain, which had arisen a few days before presentation. There was no known acute sprain or trauma, and no history of injuries in the left lower limb. The athlete localized the pain in the base of the fifth metatarsal and reported both mechanical and inflammatory pain findings. When he presented to our team, he hadn’t started any treatment for his pain. On physical examination, the ankle-foot axis was aligned, there was no edema, passive and active range of motion were normal and without instability findings, and there was no pain on resisted plantar flexion or eversion. The only finding was tenderness on the fifth metatarsal base. The athlete was a right-footed striker.

**Figure 1:**
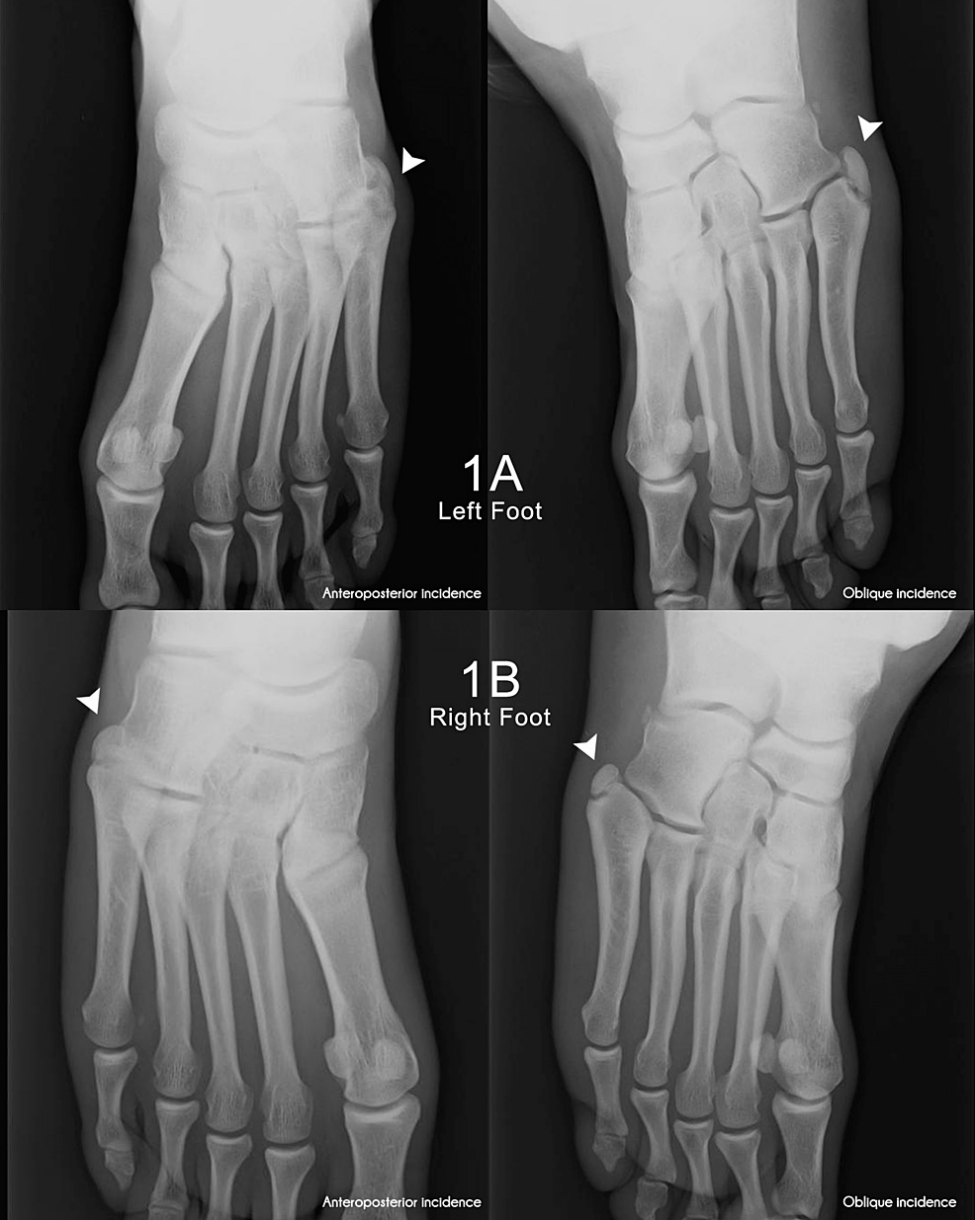
Left (1A) and right (1B) foot anteroposterior and oblique radiographs 1A Left: Anteroposterior incidence of the left foot, 1A Right: Oblique incidence of the left foot, 1B Left: Anteroposterior incidence of the right foot, 1B Right: Oblique incidence of the right foot Os vesalianum pedis is seen on all radiographs (white arrow)

We conducted a radiographic study of the left foot (Figure 1 A) with findings compatible with os vesalianum pedis. A right foot X-ray (Figure 1 B) revealed similar findings despite the absence of pain.

The athlete was treated conservatively with rest and five days of nonsteroidal anti-inflammatory drugs (NSAIDs), namely ibuprofen. He was allowed to maintain daily activities with minor impact, and we chose not to unload the left lower limb. However, any tactical, technical, endurance, or sprinting exercise with impact on the lower limbs was withdrawn for a week. The athlete was allowed muscle strengthening exercises (without impact) and static cycling. We added five days of ibuprofen 600mg twice daily. The return-to-play was seven days without any complaints of pain.

## Discussion

As described earlier os vesalianum pedis is a rare accessory ossicle and an even rarer symptomatic one [1]. Our athlete had both mechanical and inflammatory pain findings and localized the pain to the base of the fifth metatarsal. If the foot radiographs were normal, an ultrasound or even an MRI would allow a proper diagnosis. This athlete could have a peroneus brevis tendinopathy, lateral plantar fasciitis, or even a ligamentum plantare longum sprain. However, the absence of resisted eversion pain and the location of tenderness (fifth metatarsal base, instead of lateral-plantar pain) made us doubt these diagnoses. Moreover, the X-ray findings led us to other possible pathologies.

Soccer is a physically demanding sport, with a high incidence of soccer-related injuries [13]. Nonetheless, the possibility of sport-related minor traumas going unrecorded by athletes or the medical team exists [14]. Even though the athlete denied any trauma or sprain, the medical team should be aware of this possibility. When given an inattentive look at the foot radiograph, one could think that this athlete had a pseudo-Jones fracture. However, the absence of a known acute trauma and a clear fracture line would demote us from this diagnosis. The radiographic findings resemble bone consolidation with a poorly defined cortical bone. With a skeptical look, one could also argue that this athlete had a stress fracture. However, stress fractures usually occur distal to the metaphysis of the fifth metatarsal.

The literature usually describes chronic cases of symptomatic os vesalianum pedis. However, we conservatively managed our athlete with efficiency. In our opinion, either this was the initial presentation of a future chronic painful os vesalianum pedis, or the literature is biased to chronic cases, refractory to conservative treatment. It’s even possible that our athlete had a more than normal supinated heel contact, with minor trauma to peri-osseous tissues and the source of pain was not the os vesalianum pedis. However, the resolution of the short-term symptoms led us to no further diagnostic studies.

## Conclusions

Os vesalianum pedis is a rare accessory ossicle localized proximal to the base of the fifth metatarsal. Regarding foot pain, integrating the clinical and radiographic findings as well as knowledge of the accessory ossicle anatomy is mandatory to avoid misdiagnosis and mistreatment. When symptomatic, os vesalianum pedis usually presents as chronic lateral foot pain which might demand surgery. Our successful pain management indicates the possibility of early treatment as a predictor for avoiding pain chronification and, thereby the need for surgery. Thus, in symptomatic os vesalianum pedis, conservative management should always be pursued first.
